# 14-3-3θ is a Binding Partner of Rat Eag1 Potassium Channels

**DOI:** 10.1371/journal.pone.0041203

**Published:** 2012-07-20

**Authors:** Po-Hao Hsu, Shi-Chuen Miaw, Chau-Ching Chuang, Pei-Yu Chang, Ssu-Ju Fu, Guey-Mei Jow, Mei-Miao Chiu, Chung-Jiuan Jeng

**Affiliations:** 1 Institute of Anatomy and Cell Biology, School of Medicine, National Yang-Ming University, Taipei, Taiwan; 2 Brain Research Center, National Yang-Ming University, Taipei, Taiwan; 3 Graduate Institute of Immunology, College of Medicine, National Taiwan University, Taipei, Taiwan; 4 School of Medicine, Fu-Jen Catholic University, Hsin-Chuang, New Taipei City, Taiwan; University of Houston, United States of America

## Abstract

The *ether-à-go-go* (Eag) potassium (K^+^) channel belongs to the superfamily of voltage-gated K^+^ channel. In mammals, the expression of Eag channels is neuron-specific but their neurophysiological role remains obscure. We have applied the yeast two-hybrid screening system to identify rat Eag1 (rEag1)-interacting proteins from a rat brain cDNA library. One of the clones we identified was 14-3-3θ, which belongs to a family of small acidic protein abundantly expressed in the brain. Data from *in vitro* yeast two-hybrid and GST pull-down assays suggested that the direct association with 14-3-3θ was mediated by both the N- and the C-termini of rEag1. Co-precipitation of the two proteins was confirmed in both heterologous HEK293T cells and native hippocampal neurons. Electrophysiological studies showed that over-expression of 14-3-3θ led to a sizable suppression of rEag1 K^+^ currents with no apparent alteration of the steady-state voltage dependence and gating kinetics. Furthermore, co-expression with 14-3-3θ failed to affect the total protein level, membrane trafficking, and single channel conductance of rEag1, implying that 14-3-3θ binding may render a fraction of the channel locked in a non-conducting state. Together these data suggest that 14-3-3θ is a binding partner of rEag1 and may modulate the functional expression of the K^+^ channel in neurons.

## Introduction

The *ether-à-go-go* (Eag) potassium (K^+^) channel belongs to the EAG family of voltage-gated K^+^ (Kv) channels that comprises three gene subfamilies: *eag*, *erg* (*eag*-related gene), and *elk* (*eag*-like K^+^ channel) [1]. Like other Kv channels, a functional Eag K^+^ channel is a tetramer comprising four pore-forming subunits [2]. In mammals, two neuron-specific Eag subunit isoforms (Eag1 and Eag2) have been identified [3], [4], [5]. Results from *in situ* hybridization studies in rats have demonstrated that rat Eag1 (rEag1) and Eag2 (rEag2) K^+^ channel subunits are widely expressed in various regions of the brain [4], [6].

Despite their abundant expression in the brain, the neurophysiological role of Eag K^+^ channels remains obscure. One strategy to tackle this question is to identify potential Eag-interacting proteins. Epsin, a protein involved in endocytosis and cell cycle regulation, was previously shown to interact with rEag1, suggesting a functional link of the K^+^ channel to cell cycle-related signaling [7]. Calmodulin, a calcium-binding protein, has also been demonstrated to exert a calcium-dependent inhibitory effect on human Eag1 K^+^ channels [8], [9]. Interestingly, *Drosophila* Eag K^+^ channels can directly interact with Ca^2+^/calmodulin-dependent protein kinase II (CaMKII) [10], [11], which is an abundant enzyme in neurons that has been implicated to play a critical role in the modulation of synaptic plasticity [12], [13]. In addition, Camguk, a membrane-associated guanylate kinase adaptor protein that associates with CaMKII [14], was found to promote the surface expression of *Drosophila* Eag [15]. It is still unknown, however, whether CaMKII and CASK/Lin-2 (the mammalian ortholog of Camguk) may also interact with and/or modulate the biophysical properties of mammalian Eag K^+^ channels.

To further explore the potential signaling pathways associated with mammalian Eag, we set forth to identify novel binding partners of rEag1 channels in the brain. By applying the yeast two-hybrid screening of a rat brain cDNA library, we have identified 14-3-3θ as a binding partner of rEag1 K^+^ channels. In addition, we have employed biochemical, morphological, and electrophysiological assays to characterize this novel protein interaction between 14-3-3θ and rEag1 K^+^ channels.

## Materials and Methods

### Ethics Statement

All animals were handled in accordance with the National Institute of Health Guide for the Care and Use of Laboratory Animals (NIH Publications No. 80-23, revised 1996). All procedures involving animals were performed in conformity with the animal protocol approved by the Lab Animal Council, National Yang-Ming University.

### cDNA constructs

cDNAs encoding various 14-3-3 isoforms were isolated from a rat brain cDNA library (OriGene) and subcloned into a modified pcDNA3.1 vector (Invitrogen) with a myc tag. The rEag1 cDNA was kindly provided by Dr. Olaf Pongs (Institute fur Neurale, Signalverarbeitung, Zentrum fur Molekulare Neurobiologie, Germany). pSCM138 (difopein/pEYFP-C1, the 14-3-3-binding antagonist) and pSCM174 (the inactive mutant control of pSCM138.) are generous gifts from Dr. Haian Fu (Department of Pharmacology, Emory University School of Medicine, USA). All cDNAs as well as subcloned constructs were verified by DNA sequencing (Genome Research Center, National Yang-Ming University, Taiwan).

**Figure 1:**
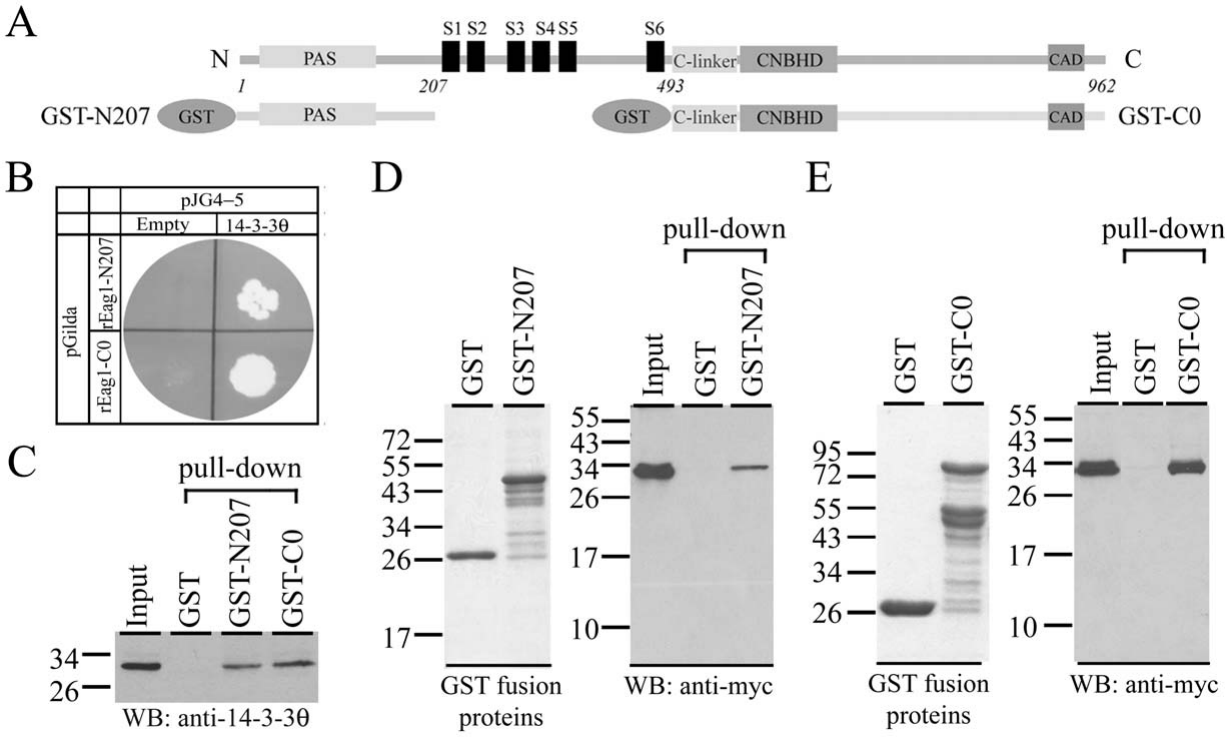
Interaction of rEag1 N- and C-termini with 14-3-3θ. *(* ***A*** *)* Schematic representation of (*top*) the structural topology of the rEag1 channel and (*bottom*) the rEag1 GST-N207 and GST-C0 fusion proteins. *(* ***B*** *)* Yeast two-hybrid assay. cDNA encoding rEag1-N207 or C0 segment was fused to the coding sequence for LexA DNA binding domain and subcloned into the pGilda vector. cDNA for the B42 transcriptional activation domain alone (*Empty*) or in combination with 14-3-3θ was subcloned into the pJG4-5 vector. Yeasts co-transformed with the pGilda- and the pJG4-5-based plasmids were streaked on leucine-lacking plates. *(* ***C*** *)* GST pull-down assay of *in vitro* translated 14-3-3θ. Pull-down products were immunoblotted with the anti-14-3-3θ antibody. Indicated to the left are the molecular weight markers (in kDa). *(* ***D,E*** *)* Cell lysates prepared from HEK293T cells expressing myc-14-3-3θ were used for GST pull-down assay with GST or the fusion protein GST-N207/GST-C0. (*Left panels*) Coomassie blue staining of the GST proteins. (*Right panels*) Immunoblotting of pull-down products with the anti-myc antibody. Input volume was 5% of that of the cell lysates for pull-down.

**Figure 2:**
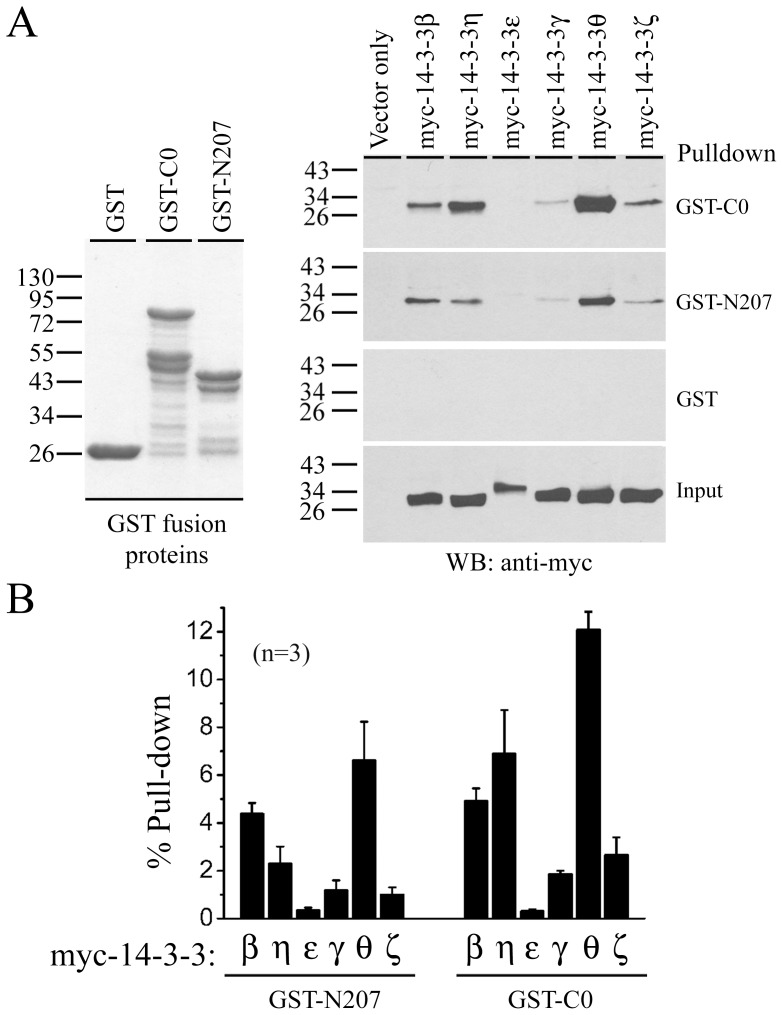
Isoform specificity of 14-3-3 binding with rEag1 N- and C-termini. *(* ***A*** *)* GST pull-down assay of cell lysates from HEK293T cells transfected with various myc-tagged 14-3-3 isoforms. (*Left panel*) Coomassie blue staining of the GST proteins. (*Right panel*) Immunoblotting of pull-down products with the anti-myc antibody. Input volume shown at the bottom corresponds to 5% of the total cell lysates for pull-down. *(* ***B*** *)* Quantification of the pull-down efficiency of different 14-3-3 isoforms. The protein band intensities of individual myc-14-3-3 isoforms affinity precipitated by GST-N207 or GST-C0 in (A) were divided by those of cognate total inputs, thereby minimizing the potential bias conferred by the variation in protein expression among different 14-3-3 isoforms. Densitometric scans of immunoblots were obtained from three independent experiments.

**Figure 3:**
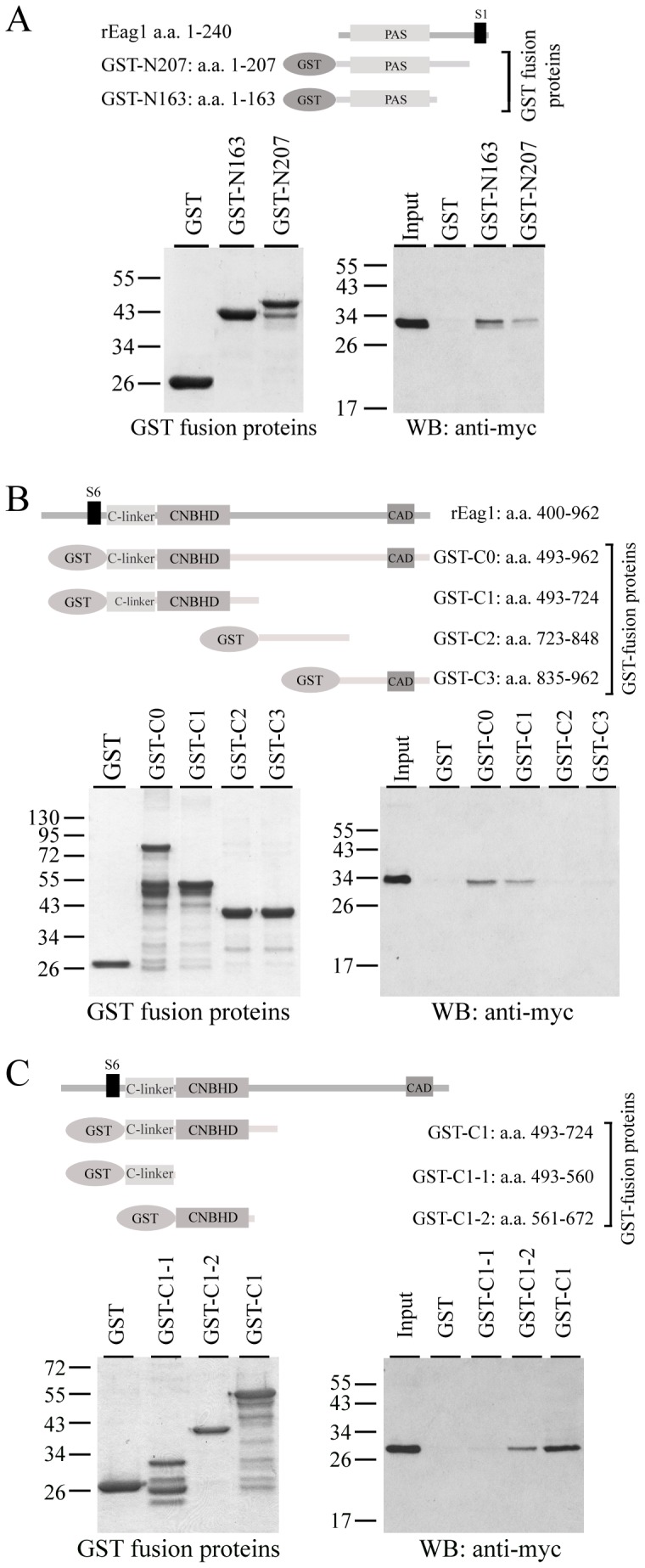
The contribution of PAS and CNBHD to rEag1 interaction with 14-3-3θ. GST pull-down assays of rEag1 N-terminal and C-terminal GST fusion proteins containing specific structural domains. (*Upper panels*) Schematic representation of the rEag1 N-terminal *(* ***A*** *)* or C-terminal *(* ***B,C*** *)* GST fusion proteins. (*Lower left panels*) Coomassie blue staining of the GST proteins. (*Lower right panels*) Immunoblotting of pull-down products with the anti-myc antibody.

### Yeast two-hybrid screening

The DupLEX-A yeast two-hybrid system (OriGene) was used to screen the rat brain cDNA library. The N-terminus of rEag1 (amino acids 1–207) was amplified by PCR and fused in-frame to the coding sequence for the DNA binding protein LexA in the yeast expression plasmid pGilda, which in turn was used as the bait to screen the library. The yeast strain EGY48, which contains the reporter gene LEU2 downstream of the LexA-operator, was sequentially transformed with *i*) the bait plasmid pGilda-N207, *ii*) the reporter plasmid pSH18-34 (containing the LexA operator-lacZ fusion gene), and *iii*) an activation domain-fused rat brain cDNA library in pJG4-5 (OriGene) by using the lithium acetate method. After incubating at 30°C for 2–7 days, transformed yeast colonies growing on leucine dropout plates were scored positive for interacting proteins. Positive colonies were further selected by the β-galactosidase assay. Plasmid DNA was extracted from yeast colonies and used to transform the *Escherichia coli* strain DH5α. Candidate cDNA clones were screened by PCR with pJG4-5-specific primers, followed by online (BLAST) and in-house sequence analyses.

**Figure 4:**
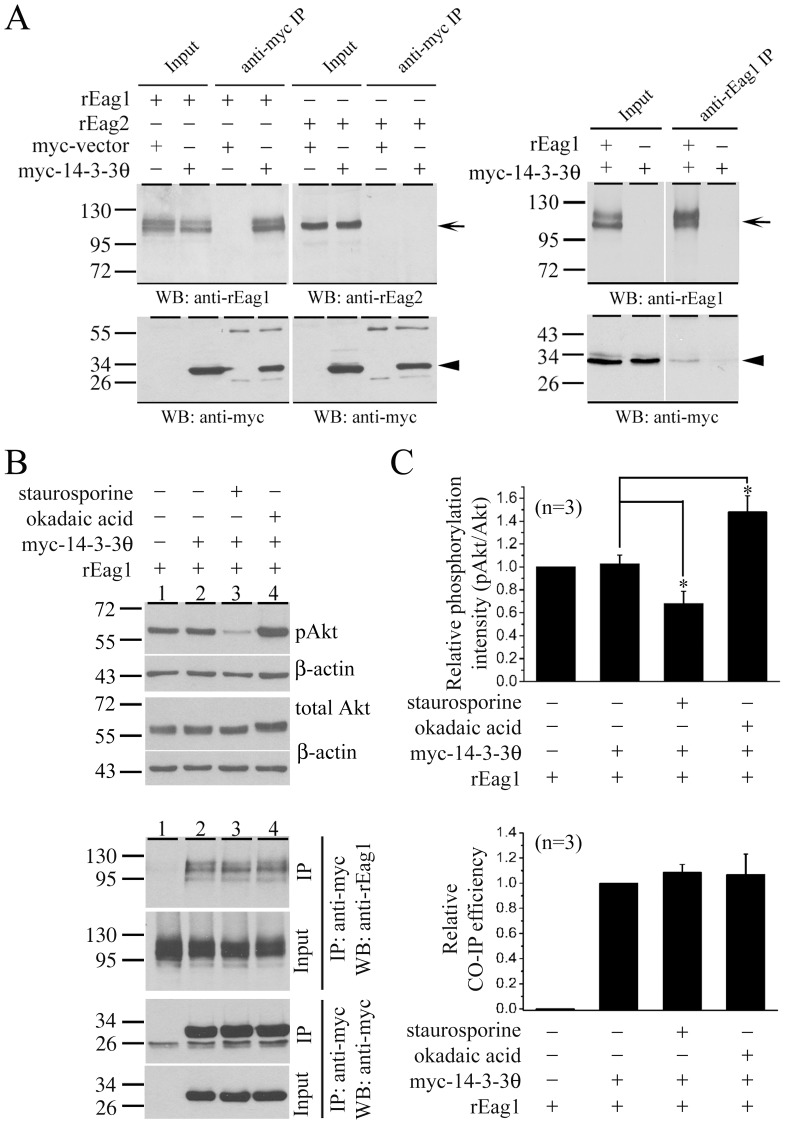
Phosphorylation-independent interaction of rEag1 with 14-3-3θ. *(* ***A*** *)* Co-immunoprecipitation of myc-14-3-3θ and rEag1 proteins. (*Left panel*) rEag1/rEag2 was co-expressed with an empty vector (*−*) or myc-tagged 14-3-3θ (*+*) in HEK293T cells. Cell lysates were immunoprecipitated (*IP*) by using the anti-myc antibody, followed by immunoblotting (*WB*) with the anti-myc or the anti-rEag1/rEag2 antibody. The protein bands corresponding to rEag1/rEag2 and 14-3-3θ are highlighted with arrow and arrowhead, respectively. (*Right panel*) Cell lysates from myc-14-3-3θ only or co-expression of rEag1 and myc-14-3-3θ were immunoprecipitated by using the anti-rEag1 antibody. Input volumes correspond to 5% of the total cell lysates used for immunoprecipitation. These co-immunoprecipitation data are representative of three to five independent experiments. *(* ***B*** *)* rEag1 was co-expressed with an empty vector or myc-tagged 14-3-3θ in HEK293T cells. 24 hrs after transfection, indicated cells were subject to 1-hr treatment with 1 µM okadaic acid or staurosporine. (*Upper panel*) Total cell lysates were immunoblotted with the anti-Akt (total Akt) or anti-phosphorylated Akt (pAkt) antibodies to monitor the cellular phosphorylation status. β-actin was run as a loading control. (*Lower panel*) Cell lysates were immunoprecipitated (*IP*) by using the anti-myc antibody, followed by immunoblotting (*WB*) with the anti-myc or the anti-rEag1 antibody. *(* ***C*** *)* Quantification of (*upper panel*) the Akt phosphorylation level (*pAkt/Akt*) and (*lower panel*) the co-immunoprecipitation (*CO-IP*) efficiency of 14-3-3θ and rEag1. The CO-IP efficiency was determined by the ratio of the protein band intensities of immunoprecipitated rEag1 to those of cognate total inputs. The mean values were subsequently normalized with respect to that of the no-treatment control of 14-3-3θ/rEag1 co-expression. Densitometric scans of immunoblots were obtained from three independent experiments. Asterisk denotes a significant difference from the no-treatment control of 14-3-3θ/rEag1 co-expression (*, *t*-test: p<0.05).

### Glutathione S-transferase (GST) pull-down assays

GST fusion proteins were produced and purified by following the manufacturer's instruction (Stratagene). In brief, the cDNA fragments encoding the rEag1 amino (N)- or carboxyl (C)-terminus were subcloned into the *Escherichia coli* expressing pGEX vector and expressed in the *Escherichia coli* strain BL21. Bacterial cultures were grown at 30°C, induced with 0.1 mM isopropyl-β-D-thiogalactopyranoside (IPTG), and then harvested by centrifugation at 8,000×*g* for 10 min at 4°C. Cell pellets were resuspended in the B-PER reagent (Pierce) containing 1 mM phenylmethylsulfonyl fluoride (PMSF) and protease inhibitor cocktail (Roche). The lysates were clarified by centrifugation at 15,000×*g* for 15 min, and glutathione-agarose beads (Sigma) were used to bind the GST fusion proteins from the supernatant. GST protein-coated beads (4–8 µg) were incubated with pre-cleared *in vitro* translated proteins or human embryonic kidney (HEK) 293 T cell lysates at 4°C overnight. The bead-protein complexes were then washed with buffer A [(in mM) 100 NaCl, 4 KCl, 2.5 EDTA, 20 NaHCO_3_, 20 Tris-HCl, pH 7.5, plus 1 PMSF, 1 Na_3_VO_4_, 1 NaF, 1 β-glycerophosphate] (with and without 1% Triton X-100), and the proteins were eluted by boiling for 5 min in the Laemmli sample buffer. *In vitro* protein translation was performed by using the TNT transcription-translation system (Promega).

**Figure 5:**
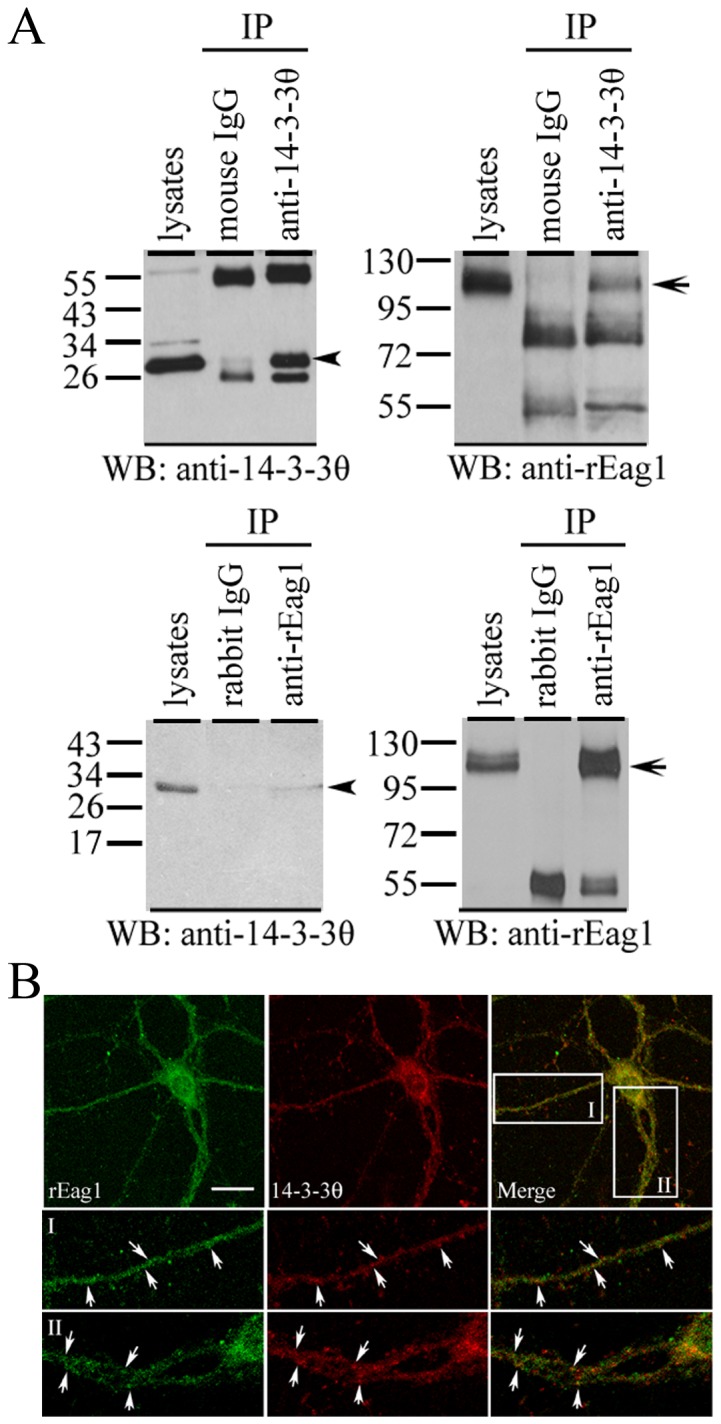
Endogenous expression of 14-3-3θ and rEag1 in neurons. *(* ***A*** *)* Co-immunoprecipitation of 14-3-3θ and rEag1. Detergent solubilized proteins from the lysates of rat forebrain were immunoprecipitated (*IP*) with the anti-14-3-3θ (*upper panel*) or the anti-rEag1 antibody (*lower panel*), followed by immunoblotting (*WB*) analyses with the anti-14-3-3θ or the anti-rEag1 antibody. The non-immune mouse or rabbit IgG was used in parallel as negative control. Input volumes correspond to 5% of the total cell lysates used for immunoprecipitation. The arrowhead and arrow refers to the protein bands of 14-3-3θ and rEag1, respectively. *(* ***B*** *)* Immunofluorescence staining of rEag1 (*left panels*) and 14-3-3θ (*middle panels*) in cultured hippocampal neurons. The area highlighted in the white boxes is viewed under a higher magnification (*I, II*). Arrows label the sites of co-localization of 14-3-3θ and rEag1 (*right panels*), which displayed significant punctuate patterns over a wide region along the neurites. Scale bar, 25 µm. These co-immunoprecipitation and immunofluorescence data are representative of four to seven independent experiments.

### Cell culture and transient transfection

Dissociated hippocampal culture and HEK293 cells stably expressing rEag1 were prepared as described previously [16]. HEK293 and HEK293T cells were maintained in DMEM (Invitrogen) supplemented with 2 mM L-glutamine, 100 units/ml penicillin/streptomycin, and 10% (v/v) fetal bovine serum (Hyclone). One day before transfection, HEK cells were grown on poly-lysine-coated coverslips. DNA transfection was performed by using the Lipofectamine 2000 reagent (Invitrogen).

**Figure 6:**
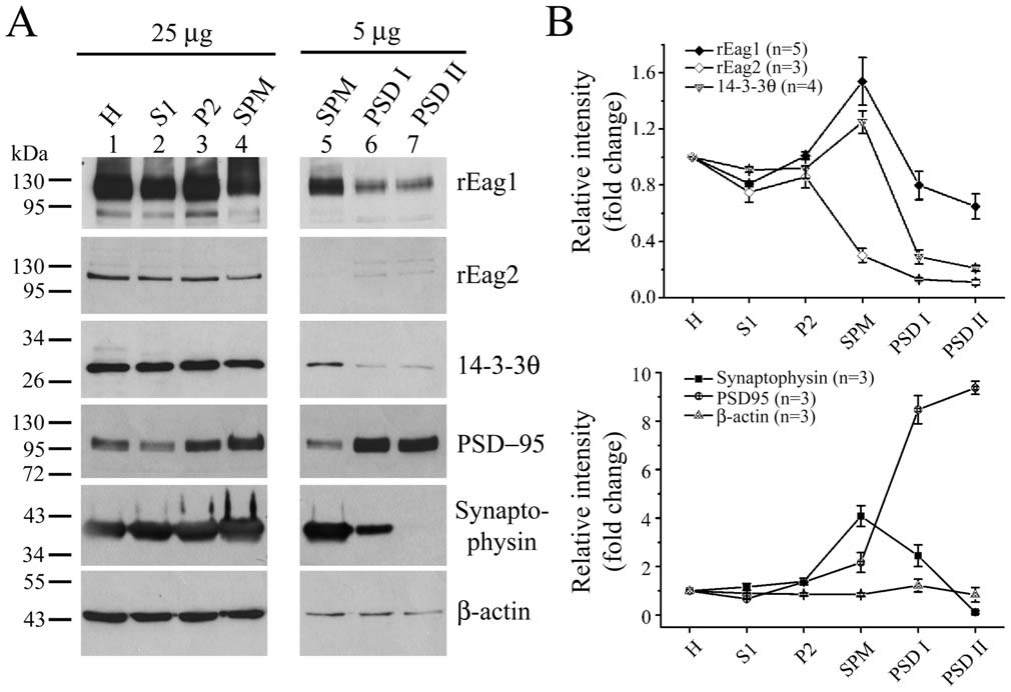
Localization of 14-3-3θ and rEag1 in synaptosomal and PSD fractions. *(* ***A*** *)* Subcellular fractionation separated rat brains into multiple fractions: homogenate (H), soluble fraction (S1), crude membrane fraction (P2), synaptosomal fraction (SPM), and two postsynaptic density (PSD) preparations (PSD I: one Triton X-100 wash; PSD II: two Triton X-100 washes), all of which were subject to immunoblotting analyses with the indicated antibodies. 25 µg and 5 µg refer to the amount of total protein loaded in each lane. *(* ***B*** *)* Quantitative analyses of protein abundance in different subcellular fractions. Densitometric scans of immunoblots were obtained from three to five independent experiments. Data were presented as normalized values with respect to cognate protein expression levels in the homogenate (H) fraction.

### Biotinylation of cell surface proteins

Cell surface biotinylation and streptoavidin pull-down was performed as described previously [17]. In brief, cells were incubated in 1 mg/ml sulfo-NHS-LC-biotin (Thermo Scientific) at 4°C for 30 min with gentle rocking. After termination and solubilization, insolubilized material was removed by centrifugation and the solubilized cell lysates were incubated for 16 hrs at 4°C with streptavidin-agarose beads (Thermo Scientific). The biotin-streptavidin complexes were eluted from the beads by boiling for 5 min in the Laemmli sample buffer.

### Co-immunoprecipitation and immunoblotting

Cell lysates were prepared by solubilizing cells in the buffer A containing 1% Triton X-100 and protease inhibitor cocktail (Roche). Insolubilized materials were removed by centrifugation. Solubilized HEK293T cell lysates or brain homogenates were pre-cleared with protein-G beads and then incubated for 16 hrs at 4°C with protein G-Sepharose (GE Healthcare Biosciences) previously coated with the indicated antibodies. After washing with ice-cold lysis buffer, the immune complexes were eluted from beads by boiling in the Laemmli sample buffer. Proteins were then separated on SDS-PAGE, immunoblotted with appropriate dilution of primary antibodies, and visualized with the ECL detection system (Western Lightning Detection Kit, PerkinElmer). Immunoblots were scanned, and protein signals were quantified by using the ImageQuant software (GE Healthcare Biosciences).

**Figure 7:**
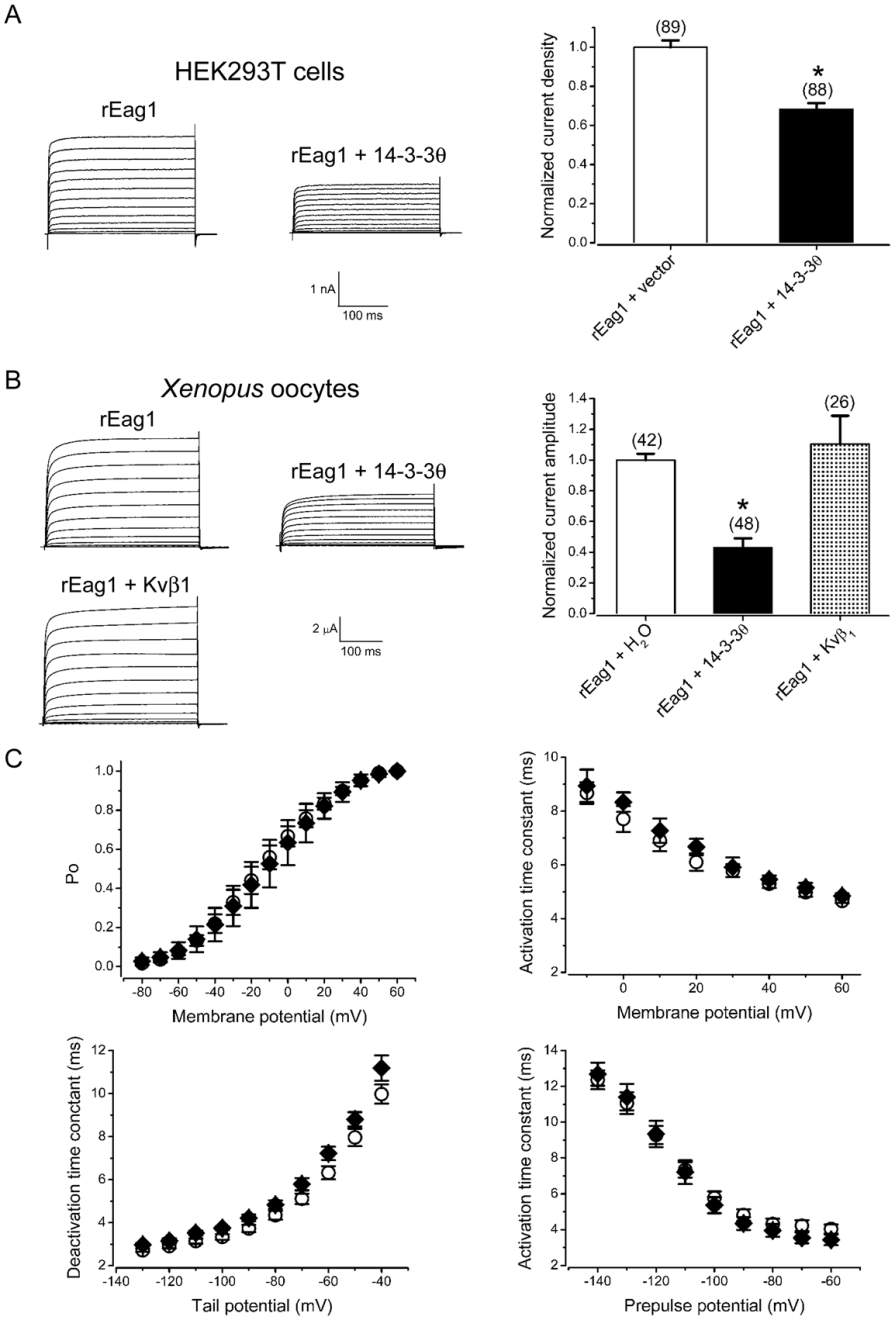
The effect of 14-3-3θ over-expression on rEag1 K^+^ currents. *(* ***A*** *)* (*Left panel*) Representative K^+^ currents recorded from HEK293T cells expressing rEag1 in the absence or presence of 14-3-3θ. HEK293T cells were co-transfected with the cDNAs for rEag1 and myc-vector or myc-14-3-3θ in the molar ratio of 1∶5. The holding potential was −90 mV. The pulse protocol comprised 300-ms depolarizing test pulses ranging from −90 to +50 mV, with 10-mV increments. (*Right panel*) Normalized mean K^+^ current density (at +40 mV) of rEag1 channels in the absence or presence of myc-14-3-3θ. The numbers in the parentheses refer to the number of cells analyzed, and the asterisk denotes significant difference from the rEag1 control (*, *t*-test: p<0.05). *(* ***B*** *)* (*Left panel*) Representative K^+^ currents recorded from oocytes expressing rEag1 in the absence or presence of 14-3-3θ. The molar ratio for cRNA co-injection was 1∶5 and 1∶10 for 14-3-3θ and Kvβ_1_, respectively. The pulse protocol was identical to that described in (*A*). (*Right panel*) Normalized mean K^+^ current density (at +40 mV) of rEag1 channels in the absence or presence of 14-3-3θ. *(* ***C*** *)* Biophysical properties of rEag1 channels in the absence (*open circles*) or presence (*filled diamonds*) of 14-3-3θ. The voltage-dependant curves for steady-state activation (*upper left panel*), activation kinetics (*upper right panel*), deactivation kinetics (*lower left panel*), and non-superimposable Cole-Moore shift (*lower right panel*) were analyzed as described previously [17]. Data were collected from recordings performed in oocytes.

**Figure 8:**
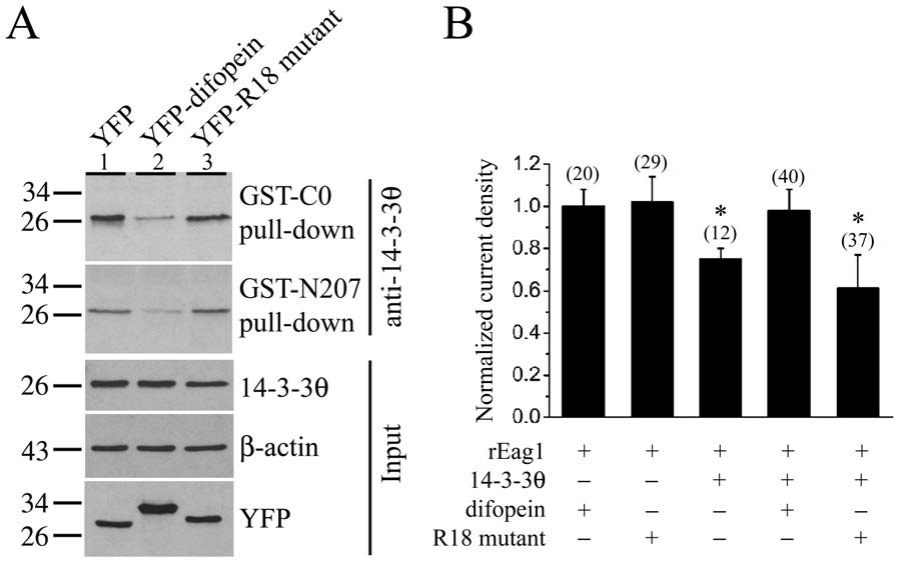
Reversal of the 14-3-3θ suppression of rEag1 K^+^ currents by the 14-3-3 antagonist difopein. *(* ***A*** *)* GST pull-down assay of the cell lysates prepared from HEK293T cells over-expressing the YFP vector, YFP-difopein, or YFP-R18 mutant. Pull-down products were detected by immunoblotting with the anti-14-3-3θ antibody. Compared to the vector control (*lane 1*), introduction of difopein (*lane 2*) resulted in a 75% and 64% reduction in the amount of 14-3-3θ pull-down by the GST-C0 and GST-N207 fusion proteins, respectively. In contrast, no significant difference was observed in the presence of the inactive mutant control (*lane 3*). *(* ***B*** *)* Normalized mean K^+^ current density recorded from HEK293 cells stably expressing rEag1 channels. As indicated, these stable cell lines were subject to transient transfection with various cDNA constructs. The mean current density at +40 mV for each co-expression condition was normalized with respect to that of the co-expression of rEag1 and difopein. The numbers in the parentheses refer to the number of cells analyzed, and the asterisk denotes significant difference from the rEag1-difopein co-expression control (*, *t*-test: p<0.05).

The antibodies used in this study include anti-14-3-3θ and anti-pan 14-3-3 (Santa Cruz Biotechnology); anti-β-actin (Sigma); anti-Akt and anti-pAkt (Cell Signaling); anti-GFP (Abcam); anti-myc (clone 9E10); anti-PSD-95 (Affinity BioReagents); anti-rEag1, anti-rEag2, and anti-Herg (Alomone Labs); and anti-synaptophysin [16].

### Immunofluorescence microscopy

Immunofluorescence staining was performed as described [16], [17]. In brief, after fixation, permeabilization, and blocking, cells were incubated overnight at 4°C in appropriate dilutions of primary antibodies (rabbit anti-rEag1 or mouse anti-14-3-3θ antibody), followed by incubation with secondary antibodies [Alexa Fluor 568 goat-anti-mouse or Alexa Fluor 488 goat-anti-rabbit antibodies (Invitrogen Molecular Probes)] at 1∶500 dilution for 1 hr at room temperature. Nuclei were labeled with DAPI. After final washes and mounting, fluorescence images of the fixed cultures were viewed with a fluorescence laser-scanning confocal microscope (Leica).

**Figure 9:**
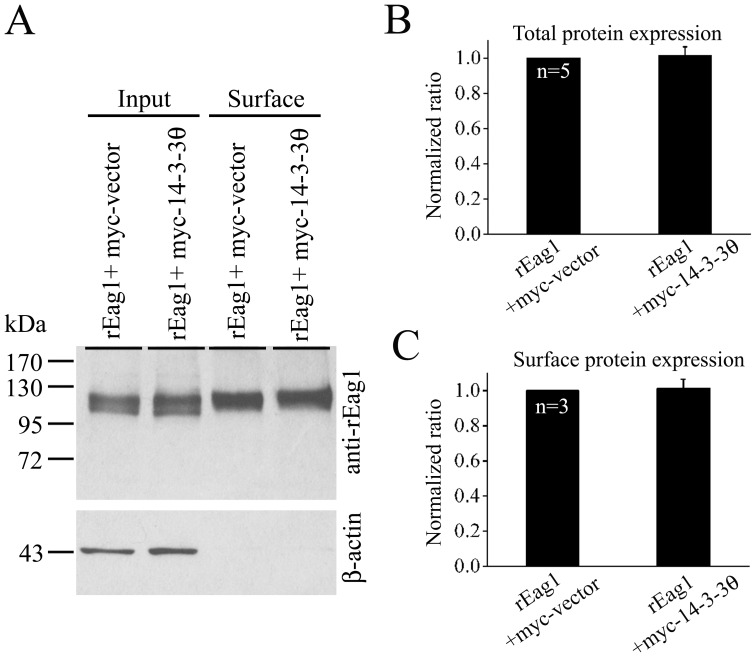
Lack of effect of 14-3-3θ over-expression on the total and surface expression of rEag1 protein. *(* ***A*** *)* Representative result of surface biotinylation experiments. Intact HEK293T cells were biontylinated on ice and thereafter solubilized. (*Surface*) Cell lysates were pulled down with streptavidin agarose beads, followed by immunoblotting with the anti-rEag1 antibody. (*Input*) Cell lysates were directly employed for immunoblotting analyses. Input represents 5% of the total protein used for streptavidin pull-down. Also shown at the bottom are the corresponding β-actin expression levels for each lane. The specificity of the biotinylation procedure was verified by the absence of β-actin bands in the surface fraction. *(* ***B*** *)* Quantification of total and surface expression of rEag1 in the absence or presence of 14-3-3θ over-expression. The total protein density (*top panel*) was determined as the ratio of input signal to the cognate β-actin signal. The surface expression efficiency (*bottom panel*) was expressed as the ratio of surface signal to the corresponding total protein density. The mean values were subsequently normalized with respect to that of vector control. Densitometric scans of immunoblots were obtained from three independent experiments.

**Figure 10:**
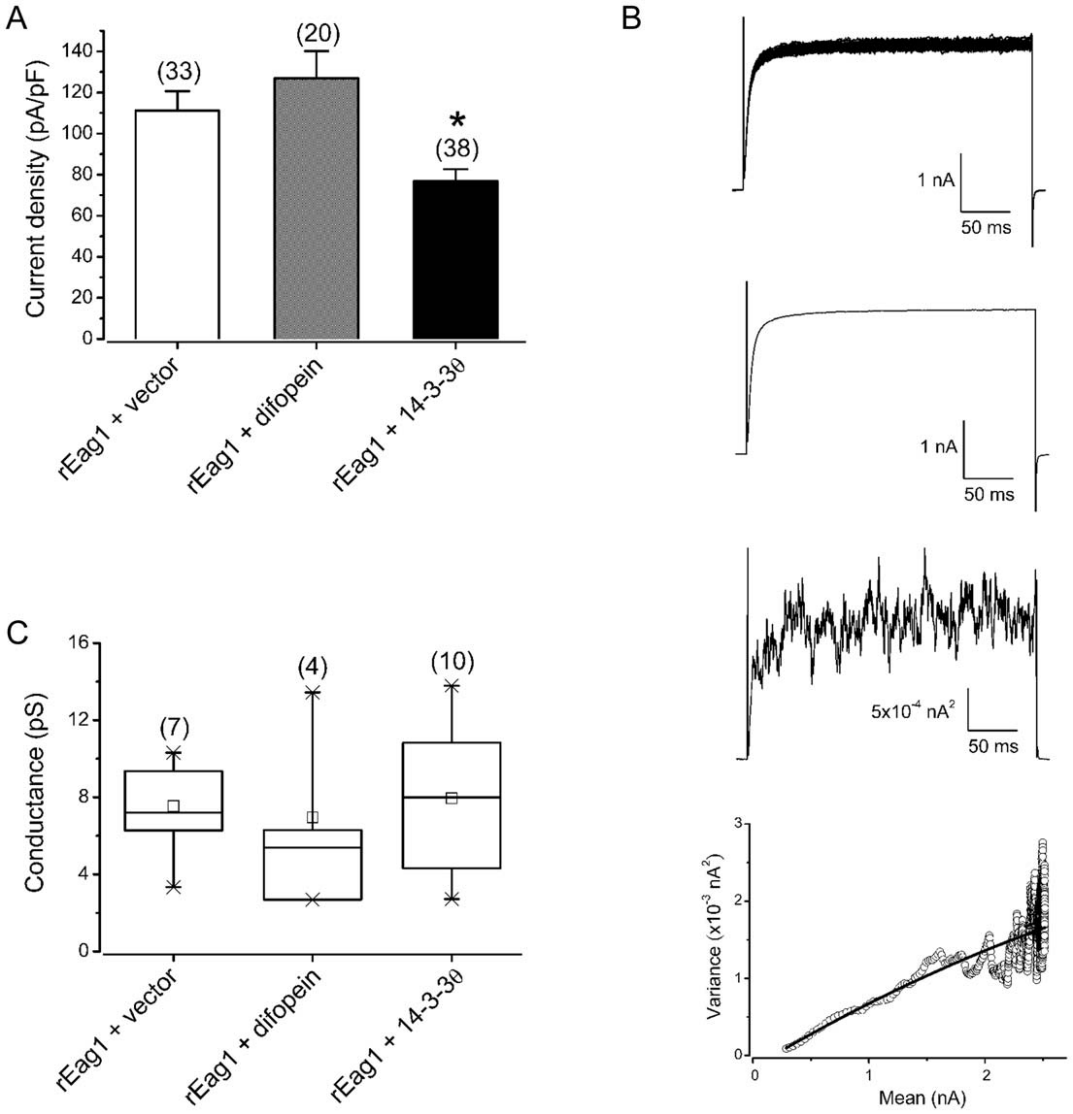
Estimation of the single channel conductance of rEag1 channel under different co-expression paradigms. rEag1 K^+^ channels were co-expressed with either the control vector, difopein, or 14-3-3θ in HEK293 cells. *(* ***A*** *)* Comparison of the mean current density (pA/pF) at +40 mV: rEag1+vector, 111.2±9.5; rEag1+difopein, 126.9±13.2; rEag1+14-3-3θ, 76.8±5.8. The numbers in the parentheses refer to the number of cells analyzed, and the asterisk denotes significant difference from the rEag1+vector control (*, *t*-test: p<0.05). *(* ***B*** *)* Representative non-stationary fluctuation analysis of rEag1 K^+^ currents. (*Top row*) Whole cell current traces were evoked by 300-ms depolarizations from −90 mV to +40 mV, which were used to generate the ensemble mean (*second row*) and variance (*third row*). (*Bottom row*) The mean-variance plot (during the 300-ms test pulse, with the linear capacitative component eliminated)(*open circles*) was fit with a parabolic function (*solid curve*) to estimate the single channel current (0.9 pA). *(* ***C*** *)* Box plot presentation of the distribution of the estimated single channel conductance. Mean values (pS): rEag1+vector, 7.5±0.9; rEag1+difopein, 7.0±2.3; rEag1+14-3-3θ, 8.0±1.1. No significant difference was found among the three co-expression conditions (One-way ANOVA: p>0.05).

### Preparation of rat brain homogenates

Rat brain tissues were homogenized with a motor driven glass-Teflon homogenizer in ice-cold dissociation buffer [(in mM) 320 sucrose, 1 MgCl_2_, 0.5 CaCl_2_, 1 NaHCO_3_, 1 PMSF and 1 mg/l leupeptin) and the cell debris was removed by centrifugation at 1,400×*g* for 10 min. The supernatant was saved, and the pellet was resuspended by homogenization in ice-cold dissociation buffer and pelleted again. The remaining pellet was discarded and the combined supernatants were pelleted (13,800×*g* for 10 min) again. The final pellet was resuspended in the buffer A containing 1% Triton X-100 and protease inhibitor cocktail.

### Subcellular fractionation of rat brains

Adult rat forebrains were homogenized in buffer H1 [(in mM) 320 sucrose, 1 NaHCO_3_, 0.5 CaCl_2_, 0.1 PMSF] containing a cocktail of protease inhibitors (Roche) and centrifuged at 1,400×*g* for 10 min to recover the supernatant S1 and the pellet P1. S1 fraction was subject to centrifugation at 13,800×*g* for 10 min to obtain the P2 pellet. The pellet was resuspended in buffer H2 [(in mM) 0.32 M sucrose and 1 mM NaHCO_3_)] and layered onto the top of the discontinuous sucrose density gradient by using 0.85, 1.0, and 1.2 M sucrose layers. The gradient was centrifuged at 65,000×*g* for 2 hrs in a Beckman Instruments SW-28 rotor and the synaptosomal fraction was recovered from the 1.0–1.2 M sucrose interface. The synaptosomal fraction was extracted in ice-cold 0.5% Triton X-100/50 mM Tris-HCl (pH 7.9) for 15 min and centrifuged at 32,000×*g* for 45 min to obtain the PSD I pellet. The pellet was resuspended and further extracted a second time with 0.5% Triton X-100/50 mM Tris-HCl (pH 7.9), followed by centrifugation at 200,000×*g* for 45 min to obtain the PSD II pellet. Protein concentration was determined by the Bio-Rad protein assay kit (Bio-Rad).

### Electrophysiology

Conventional whole-cell patch clamp technique was used to record rEag1 K^+^ currents as described previously [17]. In brief, whole-cell patch clamp was performed 24–48 hrs post-transfection of HEK293 or HEK293T cells. Patch electrodes were filled with a solution containing (in mM) 140 KCl, 1 MgCl_2_, 10 EGTA, 10 HEPES, pH 7.2. External recording solution comprised (in mM) 140 NaCl, 5 KCl, 1 CaCl_2_, and 10 HEPES, pH 7.2. Data were acquired with an Axopatch 200A amplifier (Molecular Devices) and digitized with the Digidata 1322A system and the pCLAMP 9.0 software (Molecular Devices). Cells with large currents in which voltage clamp errors might appear were excluded from data analyses. cRNA injection and conventional two-electrode voltage clamp recording of rEag1 K^+^ currents in *Xenopus* oocytes were performed as described previously [17]. In brief, stage V–VI oocytes were selected for cRNA injection. For all cRNA injection paradigms, the total volume of injection was always 41.4 nl per oocyte and the final injection amount for rEag1 cRNA was about 5 ng per oocyte. 2–3 days after cRNA injection, oocytes were functionally assayed in a recording bath containing the Ringer solution [(in mM): 115 NaCl, 3 KCl, 1.8 CaCl_2_, 10 HEPES, pH 7.2]. Voltage-clamp protocols in oocytes were applied with an OC-725C oocyte clamp (Warner) and data were digitized with the Digidata 1320A system and the pCLAMP 8.2 software (Molecular Devices). Passive membrane properties were compensated by using the -P/4 leak subtraction method provided by the pCLAMP software. Data were sampled at 10 kHz and filtered at 1 kHz. All recordings were performed at room temperature (20–22°C).

Non-stationary fluctuation analyses [18] were employed to estimate the single channel conductance of rEag1 channel from whole-cell currents (series of 40–100 traces) evoked by 300-ms depolarization from −90 mV to +40 mV. The mean (*I*) and variance (*σ^2^*) were calculated and plotted against each other, followed by curve fitting with the parabolic function: *σ^2^ = iI−I^2^/N*, where *i* and *N* represents the single channel current and the total number of channels, respectively. The estimated *i* derived from curve fitting was in turn used to calculate the single channel conductance, based on the theoretical assumption that the K^+^ equilibrium potential was −84 mV.

### Statistic analyses

All values were presented as mean ± SEM. The significance of the difference between two means was tested by Student's *t*-test, whereas means from multiple groups were compared by one-way ANOVA. All statistical analyses and curve fitting were performed with the Origin 7.0 software (Microcal Software).

## Results

### Interaction of 14-3-3θ with the N- and C-termini of rEag1 in vitro

We carried out the yeast two-hybrid screening of a rat brain cDNA library by using the N-terminus (amino acids 1–207) of the rEag1 protein (rEag1-N207) as the bait (Fig. 1A). One of the positive clones isolated by the screening was 14-3-3θ. 14-3-3 proteins are ubiquitously expressed in all eukaryotes [19]. In mammals, there are seven 14-3-3 isoforms: α/β, ε, η, γ, τ/θ, ζ/δ, and σ [20]. Except for the σ isoform, 14-3-3 proteins are abundantly expressed in the brain, and have been implicated in the modulation of neurotransmission, brain development, and learning and memory [21], [22], [23].

The potential interaction of 14-3-3θ with the N-terminus of the rEag1 protein was further validated by our yeast two-hybrid assay result showing that yeasts co-transformed with 14-3-3θ and the plasmid encoding the rEag1-N207 segment were capable of growing in synthetic leucine-lacking media (Fig. 1B). Furthermore, yeasts co-transformed with 14-3-3θ and the plasmid encoding the cytoplasmic C-terminus (amino acids 493–962) of the rEag1 protein (rEag1-C0) were also growing in the leucine-lacking medium (Fig. 1B), suggesting that 14-3-3θ may interact with the C-terminus of rEag1 as well. To address this hypothesis, we employed GST pull-down assay with GST fusion proteins encoding the N- or C-terminus of the rEag1 protein (GST-N207 or GST-C0). As depicted in Figure 1C, *in vitro* translated 14-3-3θ was efficiently retained by both GST-N207 and GST-C0 fusion proteins, but not by the GST protein *per se* (Fig. 1C), indicating a direct interaction between 14-3-3θ and the GST fusion proteins. We also performed GST pull-down assay with the cell lysates prepared from HEK293T cells transfected with myc-tagged 14-3-3θ (myc-14-3-3θ). Figures 1D and 1E demonstrate that myc-14-3-3θ was proficiently precipitated by the fusion proteins GST-N207 and GST-C0, respectively, further supporting the idea that multiple 14-3-3θ-interacting sites may exist within the rEag1 protein.

Since there are seven isoforms in the 14-3-3 protein family and some of the 14-3-3-interacting proteins have been shown to display specific binding affinity with just a subset of the 14-3-3 isoforms [24], we next asked whether the rEag1 protein exhibited a preferential interaction with specific members of the 14-3-3 protein family. Except for the σ isoform that is virtually absent in the brain, various 14-3-3 isoforms were cloned from the rat brain cDNA library and used for GST pull-down assay with the rEag1 GST fusion proteins GST-N207 and GST-C0. As illustrated in Figure 2, all but the ε isoform were precipitated by both GST-N207 and GST-C0, with 14-3-3θ showing a significantly higher binding efficiency. This observation strongly suggests that 14-3-3θ is the preferred binding partner of both the N- and C-terminal rEag1 fusion proteins.

To further address the structural basis of these novel interactions, we generated additional GST fusion proteins encoding specific protein domains of rEag1. The cytoplasmic N-terminus of rEag1 protein contains a Per-Arnt-Sim (PAS) (amino acids 14–145) domain that constitutes parts of the common structural features for the EAG K^+^ channel family [25]. To test the hypothesis that the PAS domain may contribute to the interaction between 14-3-3θ and the N-terminus of the rEag1 protein, we created another N-terminal fusion protein GST-N163 that encodes the complete PAS domain. Interestingly, Figure 3A shows that the GST-N163 fusion protein displayed a notably enhanced binding efficiency than that of GST-N207, suggesting that the PAS domain *per se* may be sufficient to mediate the N-terminal interaction of rEag1 with 14-3-3θ.

Next we switched the focus to the C-terminal interaction of rEag1 with 14-3-3θ. Since the rEag1 protein contains a long cytoplasmic C-terminus spanning over 470 amino acids, we generated three more GST fusion proteins that divided the C-terminus into three regions: GST-C1 (amino acids 492–724), GST-C2 (amino acids 723–848), and GST-C3 (amino acids 835–962). GST pull-down assay indicated that GST-C1 displayed the highest 14-3-3θ-binding efficiency, virtually identical with that of GST-C0 (Fig. 3B). This observation suggests that the proximal region of the rEag1 C-terminus may harbor a major 14-3-3θ-interacting domain. Two structural domains have been defined for this region of the rEag1 protein: the C-linker and the cyclic nucleotide-binding homology domain (CNBHD) [1], [26]. We therefore made two additional GST fusion proteins that encoded either the C-linker (GST-C1-1: amino acid 493–560) or the CNBHD (GST-C1-2: amino acids 561–672) region. As depicted in Figure 3C, 14-3-3θ preferentially bound to the GST-C1-2 fusion protein, implying that the CNBHD may contribute to the C-terminal interaction of rEag1 with 14-3-3θ.

### Interaction of 14-3-3θ with rEag1 in heterolgous and native cells

To further confirm the foregoing GST pull-down results, we tested the association of 14-3-3θ with full-length rEag1 by transiently co-expressing myc-14-3-3θ and rEag1 proteins in HEK293T cells. As shown in the left panel of Figure 4A, upon immunoprecipitating myc-14-3-3θ with the anti-myc antibody, a significant rEag1 protein signal was detected on the immunoblot. Conversely, myc-14-3-3θ was also immunoprecipitated with the anti-rEag1 antibody (Fig. 4A, *right panel*), indicating that rEag1 indeed co-existed in the same protein complex with 14-3-3θ. By contrast, no significant co-immunoprecipitation pattern was found for 14-3-3θ and rEag2, an isoform of rEag1 (Fig. 4A, *left panel*).

In most cases, 14-3-3 proteins bind to targets containing specific phosphoserine or phosphothreonine motifs [27], [28], although some non-consensus 14-3-3-binding phosphorylation motifs have been reported as well [29]. Moreover, 14-3-3 may also bind to unphosphorylated ligands [30], [31]. Upon analyzing the amino acid sequence of rEag1, we did not find any consensus or putative 14-3-3-binding phosphorylation motif. To further address this issue, we compared the effects between a phosphatase inhibitor (okadaic acid) and a protein kinase inhibitor (staurosporine). The modulatory effects of the two agents on the status of cellular phosphorylation were confirmed by the dramatic change in the phosphorylation state of the protein kinase Akt (Fig. 4B and 4C, *upper panels*). As illustrated in the lower panels of Figures 4B and 4C, neither okadaic acid nor staurosporine treatments led to noticeable change in the 14-3-3θ-binding efficiency of rEag1, consistent with idea that 14-3-3θ may interact with rEag1 in a phosphorylation-independent manner.

Given that both 14-3-3θ and rEag1 proteins are abundantly expressed in the brain, it is imperative to determine whether the potential interaction of these two proteins can also be verified in neurons. Crude membrane (P2) fractions prepared from rat forebrain homogenates were subject to immunoprecipitation with the anti-14-3-3θ antibody, followed by immunoblotting with the anti-rEag1 antibody. As demonstrated in Figure 5A, rEag1 was effectively co-immunoprecipitated with 14-3-3θ, suggesting that in the rat brain, endogenous 14-3-3θ and rEag1 co-existed in the same protein complex.

Next we asked if 14-3-3θ and rEag1 shared overlapping subcellular localization in neurons. Confocal microscopic analyses of cultured rat hippocampal neurons co-immunostained with the anti-14-3-3θ and anti-rEag1 antibodies showed that the immunoreactivities of the two proteins exhibited an extensive subcellular co-localization pattern over a broad region encompassing the soma and neurites (Fig. 5B). In addition, the rEag1 immunofluorescence displayed a notable punctate pattern over the neurites, in agreement with our previous report that rEag1 proteins may be present in the synaptic region [16]. Interestingly, in the neurites 14-3-3θ also exhibited similar punctate immunofluorescence staining.

We also compared the subcellular distribution of 14-3-3θ and rEag1 by performing the subcellular fractionation experiment. Via sucrose gradient centrifugation, the P2 fraction of rat forebrain homogenates was separated into multiple fractions, from which we collected the synaptosomal (SPM) fraction. As illustrated in the left panel of Figure 6A, both 14-3-3θ and rEag1 proteins were present in the SPM fraction, implying a co-localization of the two proteins in the synapse. Extractions with Triton X-100 further divided the SPM fraction into the postsynaptic density I (PSD I; one Triton X-100 wash) and the PSD II (two Triton X-100 washes) fractions, which provide insightful information on the subcellular localization of synapse-related proteins: the postsynaptic marker PSD-95 was highly enriched in both the PSD I and the PSD II fractions, whereas the presynaptic marker synaptophysin was only present in the PSD I fraction (Fig. 6A, *right panel*). Consistent with the aforementioned punctate co-localization patterns in cultured hippocampal neurons (see Fig. 5B), we found that rEag1 as well as 14-3-3θ were detected in both the PSD I and the PSD II fractions (Fig. 6A, *right panel*). Figure 6B provides a quantitative summary of the relative abundance of each protein in different subcellular fractions. Consistent with the co-immunoprecipitation result (see Fig. 4A), rEag1, but not rEag2, displays a similar synaptic localization profile to that of 14-3-3θ. Altogether these data provide strong evidence in support of the association of 14-3-3θ with rEag1 in the brain.

### Suppression of rEag1 K^+^ currents by the over-expression of 14-3-3θ

If the preceding inference on the protein-protein interaction is true, then is it possible that 14-3-3θ may affect the functional property of rEag1 K^+^ channels? To address this question, we studied the functional expression of rEag1 channels in the absence or presence of the over-expression of 14-3-3θ. Figure 7A exemplifies the representative result observed in HEK293T cells: over-expression of 14-3-3θ led to about 30% reduction of the amplitude of rEag1 K^+^ currents. Similarly, in *Xenopus* oocytes, over-expression of 14-3-3θ resulted in more than 50% reduction of rEag1 K^+^ currents (Fig. 7B). By contrast, the functional expression of rEag1 channels was not significantly affected by the over-expression of the auxiliary β_1_ subunit of Kv channels (Kvβ_1_) (Fig. 7B), a protein similar in size with 14-3-3θ. Other than the suppression of current amplitude, co-expression with 14-3-3θ failed to exert discernible effect on the gating properties (such as steady-state voltage dependence and gating kinetics) of rEag1 channels (Fig. 7C).

Difopein (dimeric fourteen-three-three peptide inhibitor) is a high-affinity 14-3-3 antagonist that contains two R18 peptides connected by a linker and competitively binds to all isoforms of 14-3-3, thereby effectively disrupting 14-3-3/ligand interaction in cells [32]. On the other hand, a monomeric R18 mutant in which two key residues are replaced by lysine fails to bind to 14-3-3 and hence serves as an inactive mutant control of difopein [32]. We also applied difopein and the R18 mutant to verify the functional effect of 14-3-3θ on rEag1. Figure 8A shows that the cell lysates prepared from HEK293T cells over-expressing difopein, but not the R18 mutant, significantly attenuated the amount of 14-3-3θ precipitated by both GST-C0 and GST-N207 fusion proteins, demonstrating that difopein effectively interfered with the interaction between 14-3-3θ and the N- and C-termini of rEag1. Importantly, when we co-expressed 14-3-3θ with difopein or the R18 mutant in HEK293 cells stably transfected with rEag1, the suppression effect of 14-3-3θ on rEag1 K^+^ currents was abolished in the presence of difopein, but not the R18 mutant (Fig. 8B), supporting the idea that 14-3-3θ binding is indeed indispensable for the foregoing functional modulation of rEag1 channels.

To further understand the mechanism of 14-3-3θ-induced suppression of rEag1 K^+^ currents, we asked whether co-expression with 14-3-3θ could affect the protein expression or membrane trafficking of rEag1 channels. As shown by the surface biotinylation data in Figure 9, in HEK293T cells, neither the total protein density nor the surface expression efficiency of rEag1 channels was significantly altered by the over-expression of 14-3-3θ, suggesting that the suppression effect of 14-3-3θ cannot be attributed to an alteration of the biosynthetic process of rEag1 protein.

An alternative interpretation of the functional effect of 14-3-3θ is that the molecule may reduce the single channel conductance of rEag1 K^+^ channels. To test this hypothesis, we performed non-stationary fluctuation analysis on macroscopic currents to compare rEag1 single channel conductance under different co-expression paradigms. Figure 10 shows that despite about 30% reduction in the macroscopic current density, co-expression with 14-3-3θ failed to significantly affect the estimated single channel conductance of rEag1 K^+^ channels.

## Discussion

The 14-3-3 protein family has been shown to physically interact with a wide variety of different proteins (over a hundred), including enzymes, cytoskeletal and structural proteins, kinases, membrane proteins, and transcription factors; 14-3-3 proteins may thus mediate diverse regulatory functions in many cellular processes, such as apoptosis, cell cycle, protein trafficking, and signal transduction [33], [34]. 14-3-3 proteins are also involved in the regulation of membrane ion channels [29], [35], [36]. The mechanism underlying these diverse aspects of channel regulation by 14-3-3 proteins remains elusive, as the physiological outcome of 14-3-3 modulation seems to be determined in a 14-3-3 isoform- and binding partner-specific manner. Moreover, the detailed structural basis of protein-interacting domain, as well as 14-3-3 isoform specificity, is lacking.

The results from our GST pull-down analyses indicate that the interaction with 14-3-3θ is mediated by the cytoplasmic N- and C-terminal regions of rEag1 proteins. Specifically, we propose that the N-terminal 14-3-3θ interaction site is mainly located at the PAS domain. The PAS domain is a small, modular domain found in numerous signaling molecules in both prokaryotes and eukaryotes [25]. Some of the well characterized PAS domain-dependent signaling pathways include transcriptional regulations induced by xenobiotic compounds (e.g., dioxin), circadian rhythm (e.g., CLOCK protein), or hypoxia response (e.g., hypoxia inducible factor, HIF); in most cases, the PAS core serves as the protein-protein interaction domain and may therefore determine the specificity of interactions among different signaling molecules [25], [37], [38]. Although PAS domains are found in all members of the EAG K^+^ channel family, until now their role in voltage-gated ion channels remains unclear. The current study offers a new perspective on this issue: the PAS domain may serve as a modular structural motif through which 14-3-3 proteins, and perhaps some other 14-3-3-binding proteins as well, may regulate the function of the EAG K^+^ channel family.

In addition, we propose that the C-terminal 14-3-3θ interaction site of rEag1 comprises the CNBHD, which is a conserved structural domain for the EAG K^+^ channel family, as well as cyclic nucleotide-gated and hyperpolarization-activated cyclic nucleotide-modulated channels [1], [26], [39]. The functional significance of CNBHD in the EAG K^+^ channel family is obscure, since cyclic nucleotides have been shown not to modulate Eag channels [40]. Interestingly, previous studies based on homology modeling and mutation analyses suggest that significant domain interaction may exist between the PAS domain and the CNBHD [41], which is not inconsistent with our assertion that the PAS domain and the CNBHD region constitute the N- and the C-terminal 14-3-3θ interaction sites, respectively. Further investigation will be required to determine whether one 14-3-3θ dimer *per se* could simultaneously interact with the PAS domain and the CNBHD region, which in turn may provide important structural insight into the intra- and inter-subunit organization of rEag1 K^+^ channels.

Previously, over-expression of 14-3-3ζ was shown to enhance the membrane trafficking of TASK K^+^ channels [29]. Likewise, endogenous 14-3-3 proteins promote the surface expression of ATP-sensitive K^+^ channels [42]. Our biochemical analyses, however, showed that neither the total protein expression nor the surface trafficking of rEag1 channels was reduced in the presence of over-expressed 14-3-3θ protein. Similar to our finding, it has also been demonstrated that in spite of a significant inhibition of Ca^2+^ extrusion function, over-expression of 14-3-3ε did not affect the level of protein expression or the membrane targeting of recombinant Na^+^-Ca^2+^ exchanger [43]. Moreover, 14-3-3ε was known to augment the activity of the Herg channel, another member of the EAG K^+^ channel family, by altering the channel's steady-state voltage dependence of activation [35]. In the current report, co-expression with 14-3-3θ, however, failed to exert discernible effect on steady-state voltage dependence, gating kinetics, or single channel conductance of rEag1 channels. Notably, the modulatory effect of 14-3-3ε required the phosphorylation of Herg channels [35](Fig. S1), whereas 14-3-3θ appeared to interact with rEag1 in a phosphorylation-independent manner. We therefore propose that 14-3-3θ binding may modulate the protein conformation of rEag1 such that a fraction of the K^+^ channel is virtually locked in a non-conducting closed or inactivated state. Clearly, more research is needed to understand the molecular nature of this 14-3-3θ-induced conformation change. Nevertheless, we cannot rule out the possibility that 14-3-3θ may also interact with certain rEag1-modulating factor(s) in cells, thereby indirectly down-regulating the functional expression of rEag1 K^+^ channels.

14-3-3 proteins are thought to function as U-shaped dimeric proteins, either homodimers or heterodimers, with the N- and C-terminal helices of each monomer serving as the dimerization and the ligand-binding domain, respectively [24], [44], [45]. The interaction between 14-3-3θ and rEag1 is unique in that the rEag1 protein displays a remarkable binding preference to 14-3-3θ over other 14-3-3 isoforms (see Figure 2). By contrast, TASK K^+^ channels, N-type Ca^2+^ channels, and Na^+^-Ca^2+^ exchangers virtually interact with all isoforms of 14-3-3 [22], [29], [43]. Currently, there is no clear molecular determinant available to explain ligand discrimination among 14-3-3 isoforms [24]. One potential way to address this issue in the future is to determine whether various 14-3-3θ-containing heterodimers can efficiently interact with and differentially regulate the functional expression of rEag1 K^+^ channels.

Despite their widespread expression in various regions, the precise neurophysiological functions of rEag1 K^+^ channels in the brain remain elusive. Our previous immunofluorescence studies in hippocampal and retinal neurons demonstrate that rEag1 channels display a broad localization pattern over the somatodendritic compartment, extending from somas to distal dendrites and postsynaptic membranes [16], [46]. Similarly, in the current study we found that 14-3-3θ and rEag1 exhibit significant subcellular co-localization pattern in neurons, including the synaptic region. These data are consistent with the idea that rEag1 K^+^ channels may contribute to the control of neuronal excitability over a wide range of subcellular compartment. Given our finding that 14-3-3θ suppresses rEag1 K^+^ currents, these observations raise a possibility that 14-3-3 protein may influence the functional expression of rEag1 channel in specific subcellular compartments of neurons, a plausible idea worthy of future investigation.

Since 14-3-3 proteins are abundantly expressed in the brain, it seems likely that the interaction between 14-3-3θ and rEag1 is constitutive and possesses little chance for physiological regulation. On the other hand, recent proteomic analyses of transgenic mouse brain tissues revealed that 14-3-3 proteins are associated with numerous binding partners essential for synaptic signaling and structural modulation of dendritic spines [47]; for example, 14-3-3ζ may regulate postsynaptic glutamate receptor signaling through its interaction with Homer 3, a glutamate receptor-associated scaffolding protein that is also known to form synapse protein complexes with other postsynaptic density proteins such as PSD-95 [48], [49]. Moreover, PSD-95-associated multi-protein complexes were further demonstrated to involve over a hundred proteins crucial for synaptic functions, including glutamate receptors, K^+^ channels, and scaffolding proteins [50]. It remains to be determined, therefore, whether the interaction between 14-3-3 and rEag1 may also be subject to the modulation by synaptic signaling processes involving the 14-3-3 protein family.

## Supporting Information

Figure S1 **Phosophorylation-dependent interaction of Herg with 14-3-3ε.** Herg was co-expressed with an empty vector or myc-tagged 14-3-3ε in HEK293T cells. 24 hours after transfection, cells were left untreated, or were treated with 1 µM okadaic acid or staurosporine for 60 min. *(* ***Left panel*** *)* Total cell lysates were immunoblotted with the anti-Akt (*total Akt*) or anti-phosphorylated Akt (*pAkt*) antibody. β-actin was run as a loading control. (***Right panel***) Cell lysates were immunoprecipitated (*IP*) by using the anti-myc antibody, followed by immunoblotting (*WB*) with the anti-myc or anti-Herg antibody. In the presence of the phosphatase inhibitor okadaic acid, enhanced immunopreciation efficiency was observed for Herg. By contrast, pretreatment with the protein kinase-inhibitor staurosporine led to a small but conspicuous decrease in 14-3-3ε interaction. These co-immunoprecipitation data are representative of three independent experiments.
(TIF)
