# Supplementary figures and images for: 14-3-3θ is a Binding Partner of Rat Eag1 Potassium Channels
Source: PLoS One. 2012 Jul 20;7(7):e41203. doi: 10.1371/journal.pone.0041203 (PMC3401112; doi:10.1371/journal.pone.0041203)

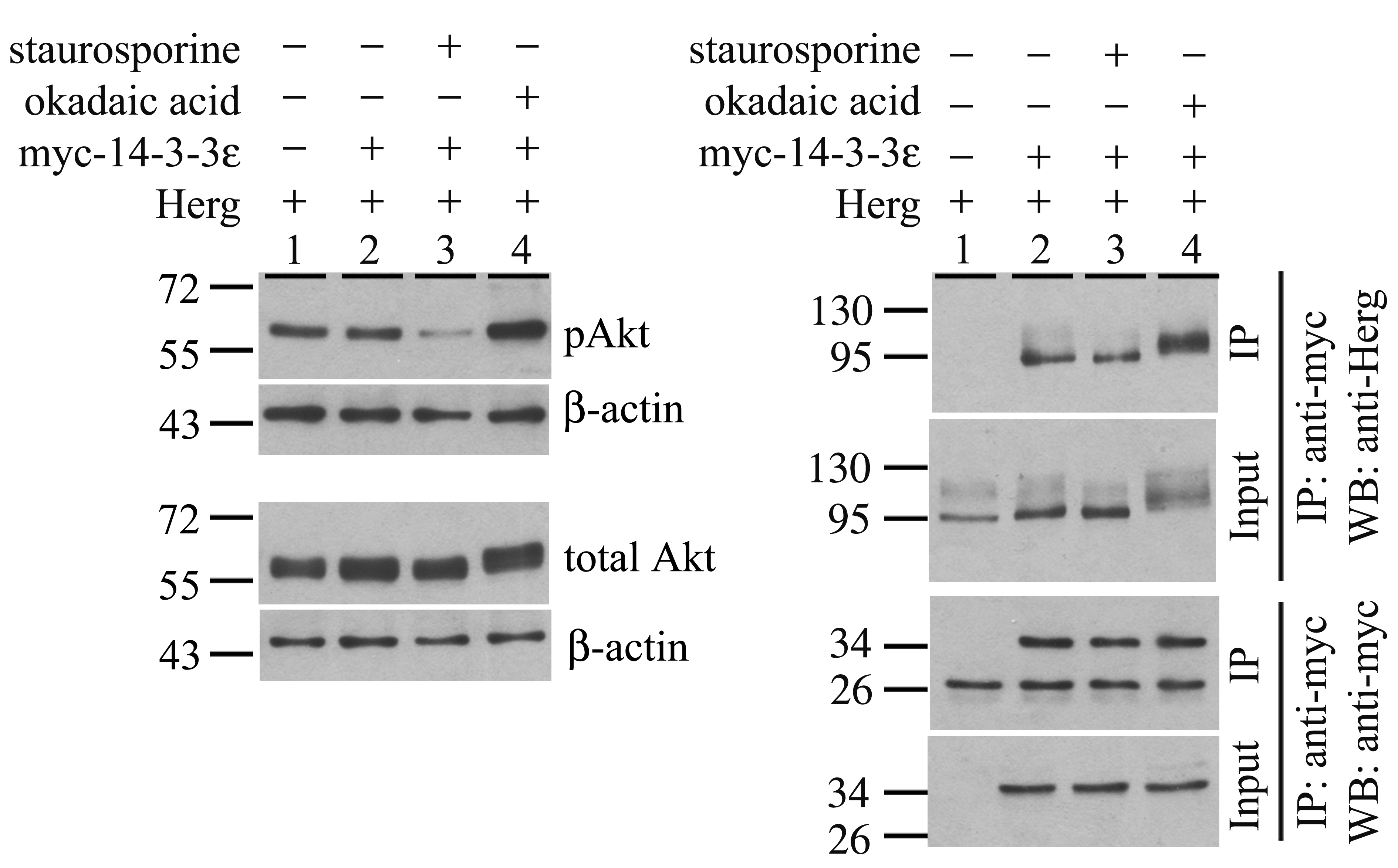

Supplement: Figure S1 — Phosophorylation-dependent interaction of Herg with 14-3-3ε. Herg was co-expressed with an empty vector or myc-tagged 14-3-3ε in HEK293T cells. 24 hours after transfection, cells were left untreated, or were treated with 1 µM okadaic acid or staurosporine for 60 min. ( Left panel ) Total cell lysates were immunoblotted with the anti-Akt (total Akt) or anti-phosphorylated Akt (pAkt) antibody. β-actin was run as a loading control. (Right panel) Cell lysates were immunoprecipitated (IP) by using the anti-myc antibody, followed by immunoblotting (WB) with the anti-myc or anti-Herg antibody. In the presence of the phosphatase inhibitor okadaic acid, enhanced immunopreciation efficiency was observed for Herg. By contrast, pretreatment with the protein kinase-inhibitor staurosporine led to a small but conspicuous decrease in 14-3-3ε interaction. These co-immunoprecipitation data are representative of three independent experiments. (TIF) [file pone.0041203.s001.tif]
